# Cerebral Perfusion and Cerebral Autoregulation after Cardiac Arrest

**DOI:** 10.1155/2018/4143636

**Published:** 2018-05-08

**Authors:** J. M. D. van den Brule, J. G. van der Hoeven, C. W. E. Hoedemaekers

**Affiliations:** Department of Intensive Care, Radboud University Nijmegen Medical Centre, Nijmegen, Netherlands

## Abstract

Out of hospital cardiac arrest is the leading cause of death in industrialized countries. Recovery of hemodynamics does not necessarily lead to recovery of cerebral perfusion. The neurological injury induced by a circulatory arrest mainly determines the prognosis of patients after cardiac arrest and rates of survival with a favourable neurological outcome are low. This review focuses on the temporal course of cerebral perfusion and changes in cerebral autoregulation after out of hospital cardiac arrest. In the early phase after cardiac arrest, patients have a low cerebral blood flow that gradually restores towards normal values during the first 72 hours after cardiac arrest. Whether modification of the cerebral blood flow after return of spontaneous circulation impacts patient outcome remains to be determined.

## 1. Introduction

The prognosis of cardiac arrest patients is mainly determined by the extent of neurological injury induced by the circulatory arrest. Return of spontaneous circulation (ROSC) does not naturally result in recovery of cerebral perfusion, as cerebral perfusion failure after ROSC is well described in animal models with no-reflow, hypoperfusion, and hyperperfusion. In animals, cerebral blood flow (CBF) ultimately restores towards normal [1]. Human studies have revealed that, in the early phase after cardiac arrest, patients have a low CBF that gradually restores towards normal values during the first 72 hours following the arrest [2–4]. In the first part of this review, the temporal course of cerebral blood flow after ROSC is described. This is relevant, because changes in cerebral blood flow can contribute to secondary brain injury. The second part of this review will focus on cerebral autoregulation after cardiac arrest, because this is an important factor in the development of ischaemia and secondary brain damage.

## 2. Cerebral Blood Flow after Cardiac Arrest

Cerebral perfusion after resuscitation is characterized by early hyperemia followed by hypoperfusion and, finally, restoration of normal blood flow. Furthermore, the blood flow is heterogeneous, with areas of no flow, low flow, and increased flow at the level of the microcirculation [5].

### 2.1. Early Hyperemia (Vasoparalysis) (0–20 min after ROSC)

Reduction of vascular tone due to tissue acidosis leads to vasoparalysis [6], which does not respond to changes in blood pressure or CO_2_ [7]. Hypoxia-induced vasoparalysis has been demonstrated in rats in the very early phase of cardiac arrest [8] and is suggested to result from an imbalance between vasodilatory and vasoconstrictive mediators in the cerebral circulation, including nitric oxide (NO) [9] and adenosine [10]. There is no direct evidence for this phenomenon* in vivo*. Hyperemia, in combination with brain swelling, can cause increased intracranial pressure, which usually normalizes before the hypoperfusion phase initiates [11]. Antioxidants and polynitroxyl albumin represent therapies that may be of value in the early hyperemia phase [12, 13].

### 2.2. Hypoperfusion Phase (20 min–12 h after ROSC)

The hypoperfusion phase is due to an impairment of the metabolic/hemodynamic coupling mechanisms, and its severity is independent of the duration of ischaemia [14]. We confirmed this lack of a relationship between ischaemia duration and the severity of hypoperfusion in comatose patients after cardiac arrest (data not published) [15]. During the hypoperfusion phase, the CBF decreases by approximately 50% [16, 17]. Several factors are implicated to play a role, including endothelial damage and an imbalance of local vasodilators (NO) and vasoconstrictors such as endothelin [18]. In this phase, impairment of the autoregulation may further decrease CBF in the setting of low blood pressure. Viable therapies for this hypoperfusion phase have been examined in animal models and include 20-hydroxyeicosatetraenoic acid inhibition by HET0016, nimodipine, and endothelin type A-antagonists [19–23].

### 2.3. Restoration of Normal Blood Flow (12–72 h after ROSC)

Finally, CBF returns to normal, remains low, or increases [24, 25]. In more recent literature, only a return to normal or an increase in CBF is described [2, 26]. Bisschops et al. described a low mean flow velocity in the middle cerebral artery (MFV_MCA_) on admission, which remained relatively stable during the first day and increased to normal levels at 48 hours [2].

The MFV_MCA_ was shown to be similar in survivors and nonsurvivors upon ICU admission [26]. However, in survivors of cardiac arrest, the MFV_MCA_ increases towards normal values in the following 72 hours, whereas a much more pronounced increase in MFV_MCA_, resulting in an overshoot of CBF, was observed in nonsurvivors [26]. This overshoot is most likely the result of a loss in vascular tone resulting in a decrease in cerebrovascular resistance in these nonsurvivors [26].

Low CBF after cardiac arrest may cause a mismatch between cerebral oxygen demand and supply. A reduction in cerebral metabolism after cardiac arrest has been described in humans and animals [27–31]. In the first 48 hours after cardiac arrest, cerebral oxygen extraction remains normal with a low CBF. This low CBF is not associated with anaerobic metabolism, determined by the jugular venous-to-arterial CO_2_/arterial-to-jugular venous O_2_ content difference ratio [32]. The jugular venous CO_2_ content significantly decreases after cardiac arrest, suggestive of low CO_2_ production due to low cerebral metabolism [32].

In survivors, the MFV_MCA_ is low immediately after cardiac arrest, accompanied by low metabolism, with a gradual restoration towards normal values accompanied by restoration of metabolism. This gradual increase of metabolism in survivors is consistent with recovery of neuronal activity. These results imply that the cerebrovascular coupling is intact in patients with a favourable neurological outcome.

In contrast, in nonsurvivors with cerebral hyperfusion, the cerebral oxygen extraction is strongly reduced, suggesting decoupling of cerebral flow and metabolism in nonsurvivors. This ongoing low metabolism likely reflects irreversible neuronal damage [32].

## 3. Cerebral Autoregulation following Cardiac Arrest

### 3.1. Cerebral Autoregulation

**Figure 1:**
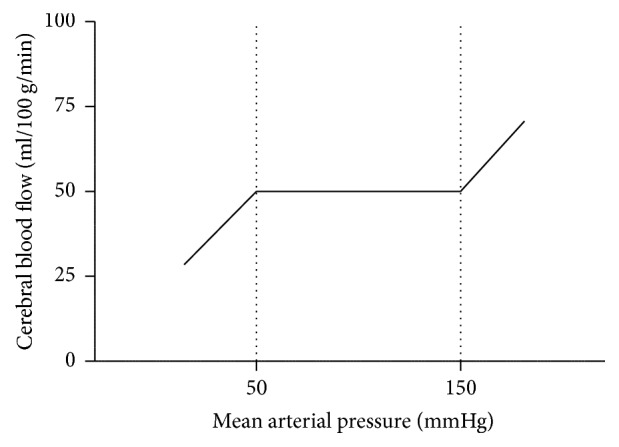
Cerebral autoregulation maintains cerebral blood flow at a constant level when the mean arterial pressure is between approximately 50 and 150 mmHg (the plateau phase).

**Figure 2:**
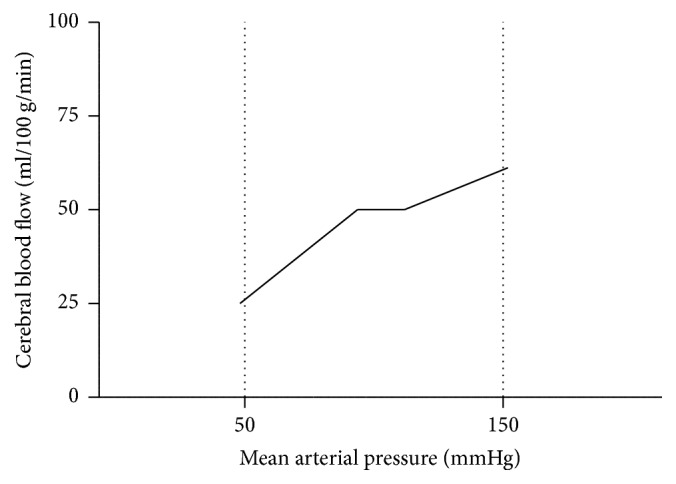
More recent data support the opinion that cerebral autoregulation does not maintain constant blood flow through a broad MAP range of 50–150 mmHg, but probably in a smaller range. Cerebral autoregulation is more effective in the range above baseline mean arterial pressure, compared to the range below.

Generally, it is assumed that cerebral autoregulation maintains CBF at a constant level when the mean arterial pressure (MAP) is between approximately 50 and 150 mmHg (the plateau phase) (Figure 1). However, more recent data suggest that cerebral autoregulation maintains constant blood flow in a smaller range [33, 34] (Figure 2). Cerebral autoregulation is more effective in the range above baseline MAP than below baseline MAP [35] (Figure 2).

The upper and lower limits of cerebral autoregulation are not fixed [36]. For example, chronic hypertension shifts these limits up. This adaptation protects the brain against high blood pressure but makes it also more vulnerable to hypoperfusion during periods of hypotension.

Dynamic cerebral autoregulation is clinically more relevant than static autoregulation, because it protects the brain against rapid alterations in blood pressure. Various methods and models are available for estimating dynamic cerebral autoregulation, using both spontaneous and induced fluctuations in blood pressure.

### 3.2. Cerebral Autoregulation following Cardiac Arrest

Cerebral autoregulation after cardiac arrest has been investigated in various studies. Initially, a linear relationship was demonstrated between MAP and CBF [37], suggesting a completely dysfunctional (static) cerebral autoregulation after cardiac arrest. Static cerebral autoregulation curves were constructed for patients after cardiac arrest by stepwise increasing MAP with vasopressors and simultaneous determination of CBF using TCD [38]. Of the 18 patients after cardiac arrest studied by Sundgreen et al., static cerebral autoregulation was absent in 8 and present in 10 patients. In five out of ten patients with preserved cerebral autoregulation, the lower limit of autoregulation was shifted upwards (range 80–120 mmHg) [38]. In fact, autoregulation may remain intact, but with a narrowed and upward shifted intact zone. This study demonstrated the heterogeneous nature of cerebral autoregulation in cardiac arrest patients.

Ameloot et al. showed that (dynamic) cerebrovascular autoregulation, determined by the moving correlation coefficient between MAP and the ratio of oxygenated versus deoxygenated hemoglobin (COX), was not preserved in one-third of postcardiac arrest patients [39]. Disturbed autoregulation was associated with unfavourable outcome [39, 40]. A MAP below the optimal autoregulatory range during the first 48 hours after cardiac arrest was associated with worse outcomes compared to patients with higher blood pressures [41].

The relationship between brain tissue oxygen saturation and MAP can also be used to determine the optimal MAP in individual patients after cardiac arrest. The feasibility of this technique to obtain real-time values for optimal MAP was demonstrated in a small prospective cohort study [42]. The optimal MAP for patients after cardiac arrest in this study was found to be 75 mmHg. In a retrospective study, Ameloot estimated the optimal MAP in patients after cardiac arrest to be 85 mmHg in patients with preserved autoregulation and 100 mmHg in patients with disturbed autoregulation [39].

Taken together, these results emphasize the importance of accurate blood pressure control in patients after cardiac arrest. Larger prospective cohort studies are required to establish the value of a tailored blood pressure targeted therapy versus conventional blood pressure targets.

The CBF changes after cardiac arrest. The critical closing pressure (CrCP) is a reliable method to quantify characteristics of the cerebrovascular bed and is defined as the lower limit of arterial blood pressure below which vessels collapse and flow ceases [43, 44]. Immediately following cardiac arrest, CrCP was shown to be high, accompanied by increased cerebrovascular resistance [26]. The CrCP decreased in the first 48 hours after admission towards normal values [26]. The CrCP was significantly higher in patients who survived compared to those who deceased [26]. Apparently, vasoactive tone was lost in patients with unfavourable outcome, resulting in reduced cerebrovascular resistance and a subsequent-increased CBF. In contrast, vasoactive tone and cerebral blood flow velocities returned to normal values in patients with favourable neurological outcome.

In addition, immediately following cardiac arrest, spontaneous variability of MFV was found to be low [15]. MFV variability increased to normal values in patients who survived, whereas it further decreased in patients who did not survive after cardiac arrest [15]. It is plausible that these changes are the consequence of the associated severe brain damage, resulting in impaired control of intrinsic myogenic vascular function and autonomic dysregulation. These changes in spontaneous fluctuations in MFV imply changes in dynamic cerebral autoregulation after ROSC.

Bisschops et al. showed a preserved cerebrovascular reactivity to fluctuations in PaCO_2_ during mild therapeutic hypothermia after cardiac arrest [2]. Previously, Yenari et al. demonstrated a preserved cerebrovascular reactivity to changes in PaCO_2_ under normothermic conditions in patients after ROSC [45]. This emphasizes the importance of strict control of blood gas values during mechanical ventilation in cardiac arrest patients, because secondary neurological damage as a result of cerebral ischaemia could be prevented by avoiding iatrogenic hypocapnia.

## 4. Conclusion

CBF is low after cardiac arrest and returns towards normal values in patients that ultimately survive. In patients with severe postanoxic encephalopathy disturbed autoregulation, loss of normal vascular tone, and increased CBF may contribute to the development of secondary brain damage, ultimately leading to fatal brain injury.

The changes in CBF after cardiac arrest may be regarded merely as a feature of severe primary brain damage resulting from ischaemia and reperfusion injury. Alternatively, they may contribute to the development of secondary brain damage. Whether modulation of the CBF after ROSC, for example, by maintaining MAP at optimal autoregulation ranges, impacts the outcome of these patients remains to be determined.

In addition, differences between CBF in the microcirculation are poorly understood and deserve more attention.
